# Effect of Exact Exchange on the Energy Landscape in Self-Consistent Field Theory

**DOI:** 10.1021/acs.jctc.4c01404

**Published:** 2025-01-17

**Authors:** Yuthika Pillai, Hugh G. A. Burton, David J. Wales

**Affiliations:** Yusuf Hamied Department of Chemistry, University of Cambridge, Lensfield Road, Cambridge CB2 1EW, U.K.

## Abstract

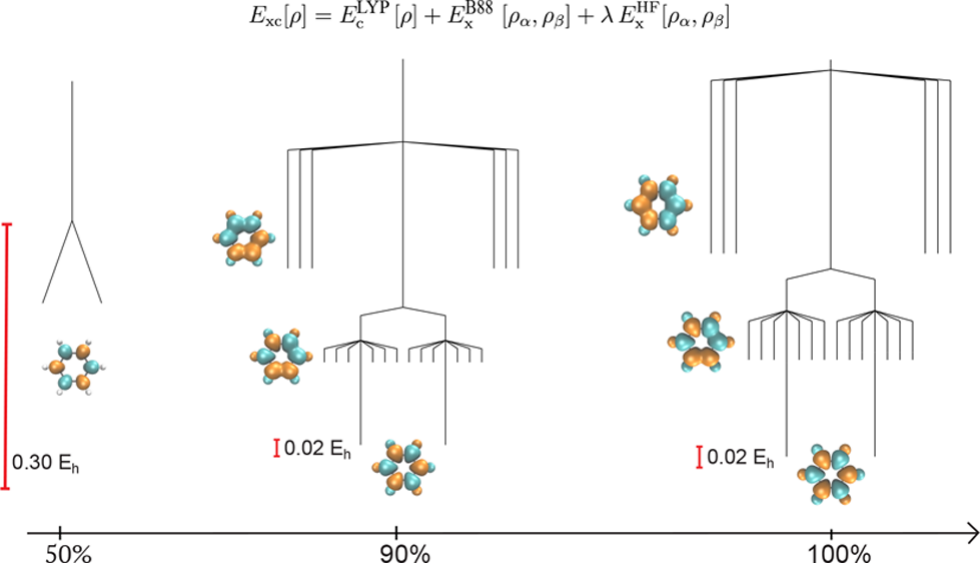

Density functional approximations can reduce the spin symmetry breaking observed for self-consistent field (SCF) solutions compared to Hartree–Fock theory, but the amount of exact Hartree–Fock (HF) exchange appears to be a key determinant in broken *Ŝ*^2^ symmetry. To elucidate the precise role of exact exchange, we investigate the energy landscape of unrestricted Hartree–Fock and Kohn–Sham density functional theory for benzene and square cyclobutadiene, which provide paradigmatic examples of closed-shell and open-shell electronic structures, respectively. We find that increasing the amount of exact exchange leads to more local SCF minima, which can be characterized as combinatorial arrangements of unpaired electrons in the carbon π system. Furthermore, we studied the pathways connecting local minima to understand the relationships between different solutions. Our analysis reveals a subtle balance between one- and two-body interactions in determining SCF symmetry breaking, shedding new light on the physical driving forces for spin-symmetry-broken solutions in SCF approaches.

## 1 Introduction

Symmetry breaking is ubiquitous in electronic self-consistent field (SCF) approximations.^1−5^ The true wave function of an isolated molecule conserves all physical symmetries of the Hamiltonian, including particle number, complex conjugation, the *Ŝ*^2^ and *Ŝ*_*z*_ spin quantum numbers *S* and *M*_*S*_, and the spatial point group symmetry. However, imposing symmetry restrictions on approximate wave functions leads to a decrease in variational flexibility, which can result in a significant increase in the variational energy. For example, in the restricted Hartree–Fock (RHF) approximation, the wave function is an eigenfunction of the *Ŝ*^2^ and *Ŝ*_*z*_ spin operators, while in the unrestricted Hartree–Fock (UHF) approximation, the wave function can provide variationally lower-energy solutions at the expense of breaking *Ŝ*^2^ symmetry (i.e., it is no longer an eigenstate of the *Ŝ*^2^ operator).^6−9^ Choosing between lower energies or conserving physical symmetries is known as Löwdin’s symmetry dilemma.^10^

Spontaneous *Ŝ*^2^ symmetry breaking is usually associated with the onset of strong electron correlation.^9^ For example, RHF fails to accurately predict potential energy surfaces for the homolytic dissociation of a chemical bond, while UHF predicts the correct dissociation energy but gives an unphysical ⟨*Ŝ*^2^⟩ value.^9,11^ The true ground state in these cases requires a multireference wave function where multiple Slater determinants provide a significant contribution. Since UHF employs a single Slater determinant, the wave function becomes symmetry-broken and “collapses” or “pins” to one of the dominant determinants in the multireference expansion. This phenomenon is known as “essential” symmetry breaking, since it reflects the deficiency of the wave function approximation, and it can be observed in bond dissociation,^11,12^ radicals,^13,14^ conical intersections,^15^ and transition metal complexes.^16−18^ Analogous features occur in the complete active space SCF approach if the active space is not large enough to capture the multireference character of the wave function.^19^

In certain cases, however, *Ŝ*^2^ symmetry breaking occurs in molecules with a ground state that is not usually considered to have strong static correlation.^20^ For example, benzene has a stable closed-shell electronic structure, suggestive of a single dominant reference configuration, but the UHF global minimum breaks *Ŝ*^2^ symmetry at the equilibrium geometry.^21^ These cases can be considered as “artificial” symmetry breaking, since a single Slater determinant provides a qualitatively correct representation of the true ground state.^1,20^ Instead, various authors have attributed artificial symmetry breaking to a lack of dynamic correlation, since a symmetry-pure wave function can be recovered by optimizing the orbitals in the presence of perturbative corrections.^14,20,22,23^

Identifying the origins of artificial symmetry breaking, and developing techniques to mitigate against it, is vital to avoid subsequent issues such as incorrect potential energy surfaces or molecular properties.^22^ Previous work has shown that this effect can be removed by optimizing the orbitals in the presence of some approximate electron correlation, using Kohn–Sham (KS) density functional theory (DFT)^13^ or Møller–Plesset theory.^20^ Furthermore, this symmetry breaking is known to be sensitive to the choice of DFT exchange–correlation functional.^13,14^ In particular, ref (13) showed that including a greater amount of HF exchange in hybrid functionals, such as B3LYP, can increase the severity of symmetry breaking. On the other hand, a smaller amount of HF exchange means that hybrid functionals are less effective at overcoming the self-interaction error.

While it is known that exact exchange can increase the tendency for orbitals to break spatial and spin symmetry using density functional approximations, the full relationship between this exchange interaction and the electronic structure of symmetry-broken solutions remains obscure. For example, why does the UHF ground state in benzene form an antiferromagnetic structure and are other types of configuration possible? Furthermore, the effect of exact exchange on the underlying SCF energy landscape has yet to be determined, such as the number of local minima and the connectivity of the different solutions. Elucidating this SCF landscape structure is important for understanding how artificial and essential symmetry breaking and the choice of density functional approximation determine the reliability and efficiency of numerical SCF calculations.

In this contribution, we develop a better understanding of how the HF exchange influences artificial and essential SCF symmetry breaking by analyzing the organization of the electronic energy landscape.^24,25^ This landscape perspective provides a more complete picture of the solution space for SCF theory by revealing the connectivity of different local minima, moving beyond a standard analysis of the solutions themselves. Furthermore, it allows us to systematically and exhaustively identify *all* local minima of the SCF energy, revealing how the choice of the functional or molecular structure affects the complexity of the solution space. We investigate the SCF energy landscapes for benzene and square cyclobutadiene using HF and DFT functionals, systematically varying the amount of exact HF exchange. Our results demonstrate the intimate relationship between HF exchange and artificial SCF symmetry breaking, revealing intuitive patterns of spin-polarized solutions that emerge from a fine balance between electron delocalization and the two-electron exchange interaction.

In Section 2, we summarize the computational procedure employed to explore the SCF energy landscape. Section 3 provides a detailed discussion of our numerical results for benzene and cyclobutadiene. Our key findings and conclusions are outlined in Section 4.

## 2 Theoretical Details

### 2.1 Differential Geometry of SCF Theory

In unrestricted Kohn–Sham density functional theory (KS-DFT),^26^ the ground state energy is expressed using a functional of the density ρ as
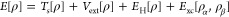
1where *T*_*s*_[ρ] is the kinetic energy of a system of non-interacting electrons, *V*_ext_[ρ] is the external (electron–nuclear) potential, *E*_H_[ρ] is the Coulomb interaction, and *E*_xc_[ρ_α_, ρ_β_] is the exchange–correlation functional. In principle, this formalism allows the full many-electron system to be mapped to a fictitious system of noninteracting particles where the wave function corresponds to a single Slater determinant, assuming that the exact exchange–correlation functional is known. In an unrestricted formalism, the high-spin (α) and low-spin (β) electrons may occupy different spatial orbitals, ϕ_*p*_^α^(***r***) and ϕ_*p*_^β^(***r***), which can be represented in terms of *n* (nonorthogonal) basis functions χ_μ_(***r***) as^9^
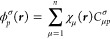
2where σ ∈ {α, β}. In this work, we consider only real-valued orbitals with , and we use the indices *i*, *j*, *k*, ... for occupied orbitals, *a*, *b*, *c*, ... for virtual orbitals, and *p*, *q*, *r*, ... for arbitrary orbitals. The total one-particle density for a system with *N*_α_ + *N*_β_ electrons is then defined as
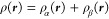
3where the spin components are defined as
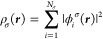
4and an exact expression for the noninteracting kinetic energy in atomic units is given as
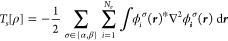
5The HF approximation is recovered by equating the exchange–correlation functional *E*_xc_ to the exact exchange, defined in terms of the orbitals as
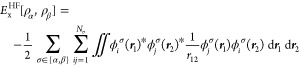
6

Self-consistent solutions to the KS-DFT or HF equations correspond to stationary points of *E*[ρ] with respect to the orbital coefficients *C*_μ*p*_^σ^, subject to the orthogonality constraint
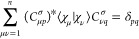
7Since the energy and density are invariant to occupied–occupied or virtual–virtual orbital rotations (orthogonal transformations), this optimization is constrained to a Grassmann manifold.^27,28^ Variations in the orbital coefficients on each SCF iteration *k* can then be parameterized using an exponential Thouless transformation^29−31^ for each spin component as
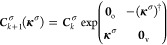
8where **κ**^σ^ is an (*n* – *N*_σ_) × *N*_σ_ matrix containing rotation angles between the virtual and occupied orbitals and the notation **0**_o_ or **0**_v_ denotes an *N*_σ_- or (*n* – *N*_σ_)-dimensional identity matrix, respectively. The constrained gradient includes two components corresponding to variations in the α and β orbitals, which are coupled through the nonlinear Fock operator *F̂* and are defined individually by the virtual–occupied block as^24,30,32,33^
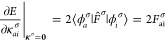
9The σ component of the Fock operator is defined self-consistently as
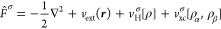
10and the Coulomb and exchange–correlation potentials are defined as the functional derivatives
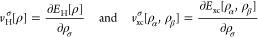
11Since the energy is invariant to rotations among the occupied or virtual orbitals, the stationary condition *F*_*ai*_^σ^ = 0 is equivalent to the standard self-consistent eigenvalue formulation
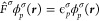
12where ϵ_*p*_^σ^ are the single particle orbital energies.

### 2.2 Optimization Techniques

Following our previous work in ref (24), we systematically search for SCF stationary points through an interface between Q-Chem and the OPTIM program, providing access to a variety of numerical optimization techniques. Local minima were located using a customized L-BFGS routine^34−38^ in OPTIM.^39^ The index-1 saddle points that connect different minima (transition states^40^) were located using gradient-only hybrid eigenvector-following,^41−44^ following systematic perturbing steps **κ** applied to the molecular orbital coefficients corresponding to local minima. Approximate steepest-descent paths were calculated to establish the connectivity using L-BFGS minimization following small displacements parallel and antiparallel to the eigenvector corresponding to the unique negative Hessian eigenvalue. Distinct solutions were distinguished using the density distance metric introduced previously,^24^ defined for two Slater determinants |Φ_*x*_⟩ and |Φ_*y*_⟩ as
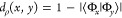
13which is bound in the range *d*_ρ_(*x*, *y*) ∈ [0, 1].

To accelerate the search for all local minima and index-1 saddle points, we periodically search for symmetry-related copies of known stationary points using the symmetry operators *R̂* ∈ Ω ⊗ *T* belonging to the direct product group of the spatial point group Ω and the time-reversal group .^24^ For real unrestricted orbitals, the time-reversal operator τ̂ corresponds to the spin-flip operation. Once one solution in each degenerate set has been identified, |Φ_*x*_⟩, every other solution within that set can be found through a symmetry operation . These operations also interconvert symmetry equivalent steepest-descent pathways and thus determine the connectivity between symmetry-related minima and index-1 saddle points. We implemented this systematic search using the poly-inspect library^45^ to generate symmetry-related initial guesses from known solutions, which were then verified as true stationary points in OPTIM.

### 2.3 Disconnectivity Graphs

The organization of multidimensional potential energy surfaces, such as the SCF energy landscape, can be visualized using disconnectivity graphs.^46,47^ Each vertical branch in the graph represents a local minimum and terminates at the corresponding energy on the vertical axis. A node connects two lines at the lowest energy for which the two minima can be interconverted via index-1 saddle points, determined at a set of regular energy thresholds. If multiple pathways connect two minima, then only the lowest-energy pathway affects the disconnectivity graph. The resulting graph then reveals the organization of local minima into disjoint sets of minima, where the members of each set can interconvert below the given energy threshold (superbasins), providing a graphical representation of the energy landscape that preserves the barriers between minima and avoids any low-dimensional projection. The minima are usually organized on the horizontal axis so that the lowest members of each group lie in the middle.

## 3 Results and Discussion

### 3.1 Artificial versus Essential Symmetry Breaking

**Figure 1:**
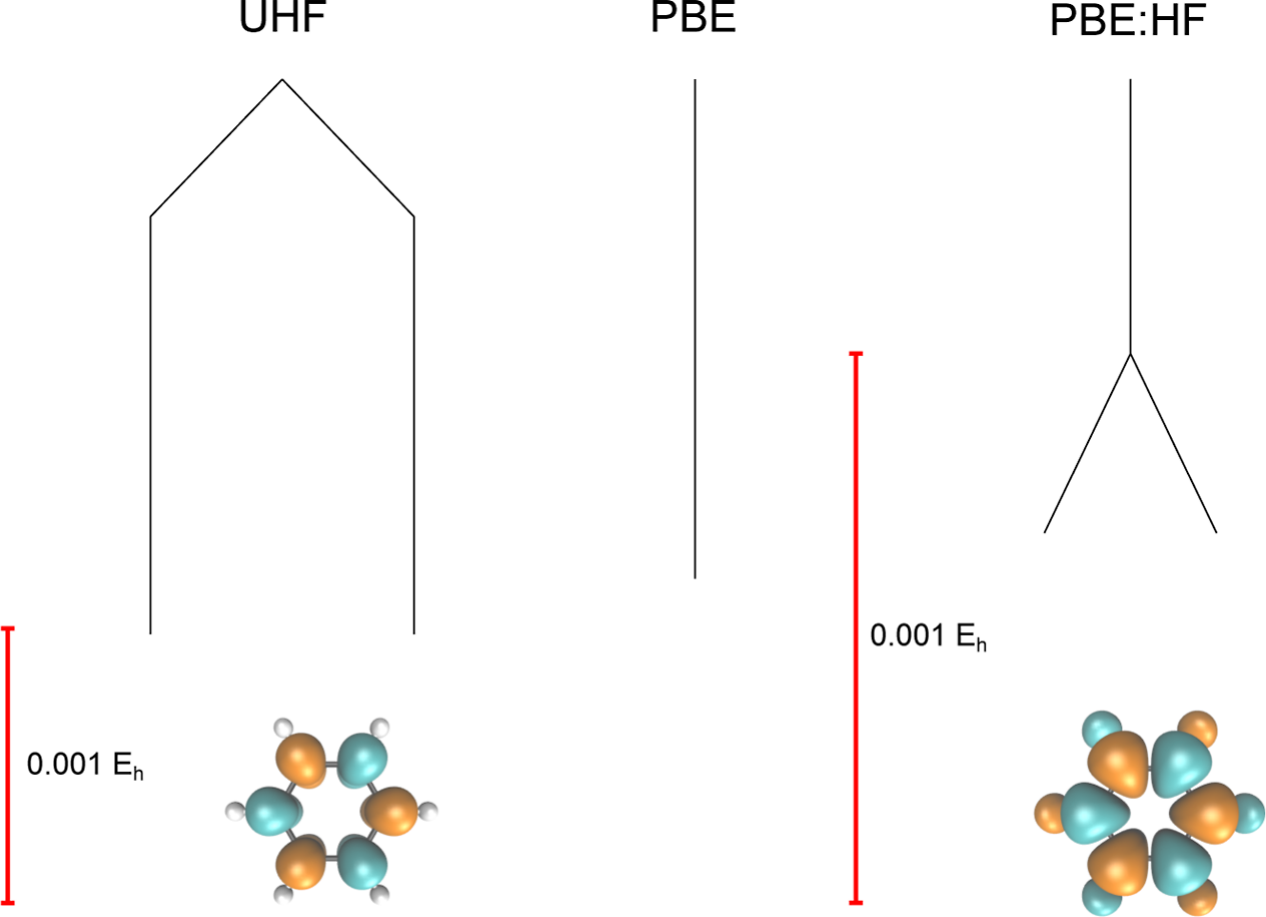
Comparison of the UHF and UKS energy landscapes for benzene using pure HF, pure PBE, and a combination of the PBE correlation functional with the HF exchange functional (PBE:HF). The UHF global minimum breaks *Ŝ*^2^ symmetry with an antiferromagnetic spin density (as shown), forming a pair of degenerate minima that are related by a global spin reversal. This artificial symmetry breaking is removed by the PBE exchange functional. A molecular structure close to the equilibrium geometry (see Supporting Information) was employed along with a 6-31G* basis set.

We start by assessing the effect of the exchange interaction on the SCF solutions at the equilibrium geometry of benzene (provided in the Supporting Information). For UHF with the 6-31G* basis set,^48^ there are two degenerate minima that represent the ground state (Figure 1). These solutions break spin symmetry with  and have an antiferromagnetic spin pattern, as shown for one of the minima in the spin density plot in Figure 1. The degenerate minima are related by a spin reversal of all the spins and are connected by an index-1 saddle point that corresponds to the RHF ground state, with .

This symmetry breaking can be considered artificial because benzene is expected to have a closed-shell electronic structure and thus should not feature the (poly)radical character that arises on spin symmetry breaking. To rule out the possibility that this effect is due to the basis set, we also analyze the UHF energy landscape with the 3-21G^49^ and cc-pVDZ^50^ basis sets (Table 1). We obtain the same landscape structure, with two degenerate minima for all the basis sets considered, confirming that the symmetry breaking is a consequence of the wave function approximation or choice of functional rather than the basis set.

**Table 1:** Enumeration of Minima of the SCF Energy Landscape for Benzene

basis set	theory	minima
3-21G*	HF	2
	TPSS^51^	1
	PBE^52^	1
6-31G*	HF	2
	TPSS	1
	B3LYP^53,54,55^	1
	PBE	1
	B97-D3(BJ)^56,57^	1
	M06-2X^51^	1
	MGGA_MS1^58^	1
cc-pVDZ	HF	2
	TPSS	1

To investigate whether this artificial symmetry breaking is affected by including approximate electron correlation using DFT, we analyzed the energy landscapes for a variety of meta-GGA and hybrid functionals (Table 1). In each case, there is only one minimum, corresponding to a RKS solution with . This simple energy landscape with a single minimum is illustrated for the PBE functional as a single vertical branch in Figure 1, where artificial symmetry breaking is removed. Furthermore, the number of transition states is also reduced with the PBE functional. This reduction in the total number of stationary points suggests that the SCF energy landscape for these functionals is smoother than the HF landscape, consistent with previous results.^24^

Previously, ref (13) suggested that the primary driving force for spin symmetry breaking in DFT is the inclusion of HF exchange, rather than the choice of correlation functional. To understand how exact HF exchange affects the SCF energy landscape, we rescale the exchange functional between pure PBE and pure HF using a custom exchange–correlation functional defined as
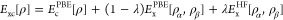
14where λ ∈ [0, 1]. In contrast to the pure PBE functional, which has a single spin-pure global minimum, increasing the amount of HF exchange creates a pair of degenerate spin-polarized minima that are analogous to the UHF global minima. These symmetry-broken solutions emerge for higher values of exact exchange. The corresponding SCF energy landscape at λ = 1 is labeled PBE:HF in Figure 1. The structure of the SCF energy landscape using the PBE correlation functional with pure HF exchange is isomorphic to the corresponding landscape using the HF approximation, demonstrating the crucial role of HF exchange in driving spin symmetry breaking in benzene.

**Figure 2:**
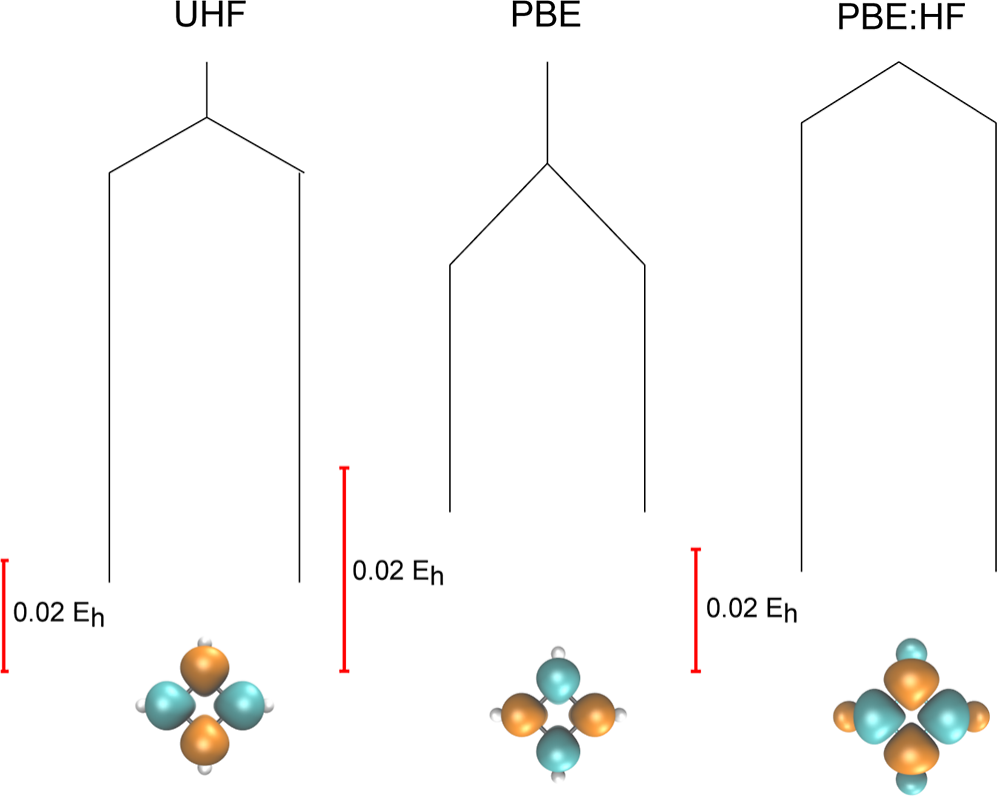
Comparison of the UHF and UKS energy landscapes for square cyclobutadiene using pure HF, pure PBE, and a combination of the PBE correlation functional with the HF exchange functional (PBE:HF). The global UHF minimum breaks *Ŝ*^2^ symmetry with an antiferromagnetic spin density (as shown for one of the minima), and this essential symmetry breaking also occurs for the PBE exchange functional. A molecular structure close to the equilibrium geometry (see Supporting Information) was analyzed using the 6-31G* basis set.

In contrast to benzene, square cyclobutadiene is an antiaromatic molecule with a diradical open-shell character. Therefore, it is expected that essential symmetry breaking will occur for a single determinant approximation. Using the UHF approximation near the equilibrium geometry, there are two degenerate UHF minima with  (Figure 2). These global minima also have an antiferromagnetic spin-polarization pattern that is analogous to the UHF global minima in benzene. However, unlike benzene, the symmetry-broken solutions continue to exist for the PBE exchange–correlation functional, both with and without exact exchange, as shown for the PBE functional in Figure 2. The persistence of these spin-polarized solutions when approximate correlation is included with a density functional approach is consistent with the assignment of essential symmetry breaking, as expected for the diradical ground state in square cyclobutadiene.

### 3.2 Introducing Excess Exchange Contribution

**Figure 3:**
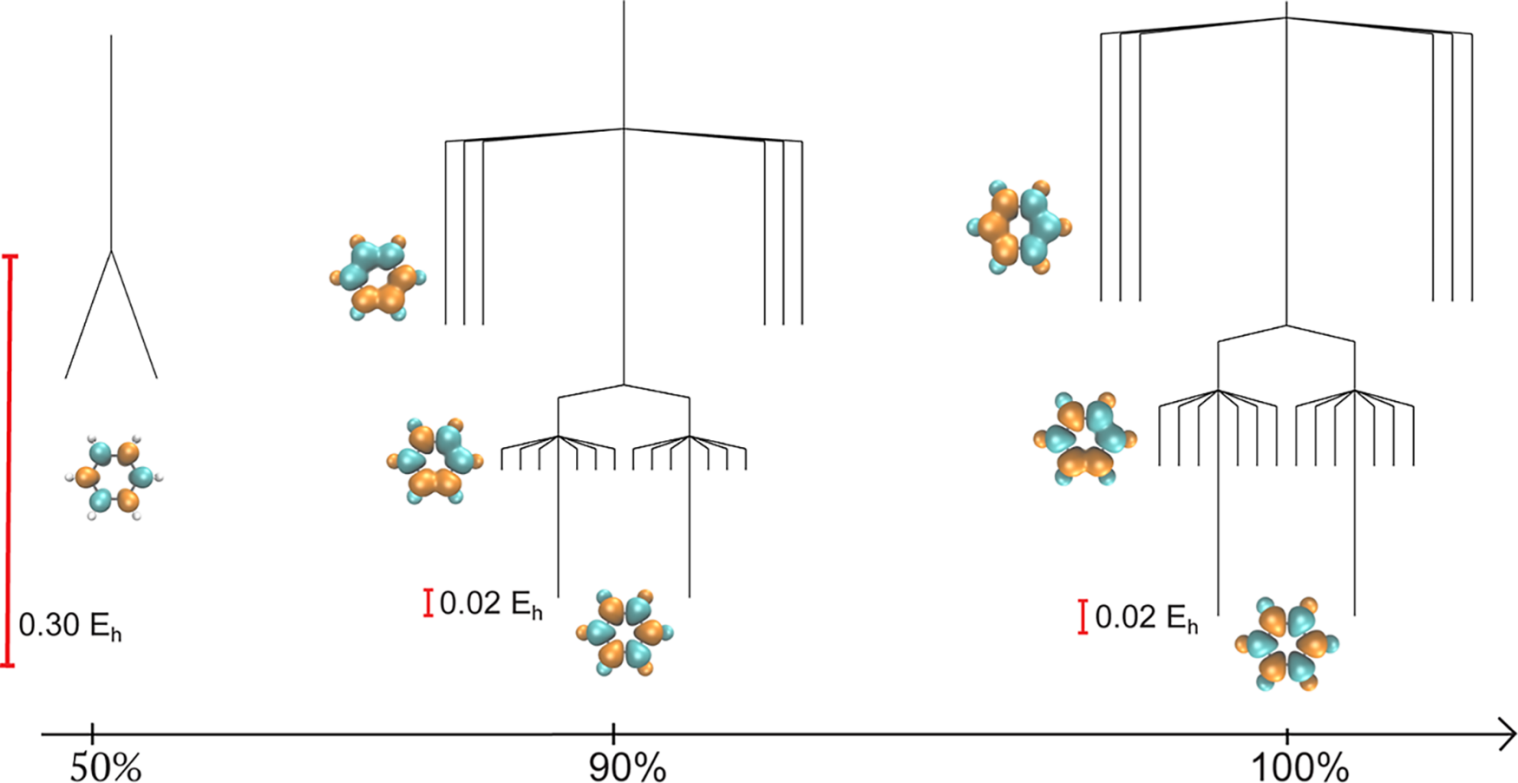
Evolution of the SCF energy landscape for benzene (6-31G*) using the custom functional defined in eq 15. Additional spin-polarized local minima emerge as the amount of HF exchange is increased, providing further evidence that the exchange interaction drives spin symmetry breaking. Selected minima are visualized with spin density plots.

To further investigate the effect of the exchange interaction on the SCF energy landscape, we introduce surplus exchange using a custom functional of the form
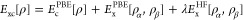
15where λ ∈ [0, 1]. At λ = 1, this combined functional includes an equal mixture of PBE and HF exchange and the total exchange interaction is intentionally double counted. This addition of surplus exchange exaggerates the spin symmetry breaking in the SCF energy landscape, as illustrated for benzene in Figure 3. As the amount of HF exchange included on top of the PBE functional increases, additional local minima emerge at an increasingly higher energy, with a total of 20 local minima for 100% HF exchange. The appearance of these higher-energy minima indicates that exchange interactions drive the appearance of symmetry-broken minima for identical PBE correlation contributions. Crucially, including an excess exchange interaction results in a more complicated energy landscape, with obvious implications for the efficiency of global optimization and applications of SCF methods in general.

**Figure 4:**
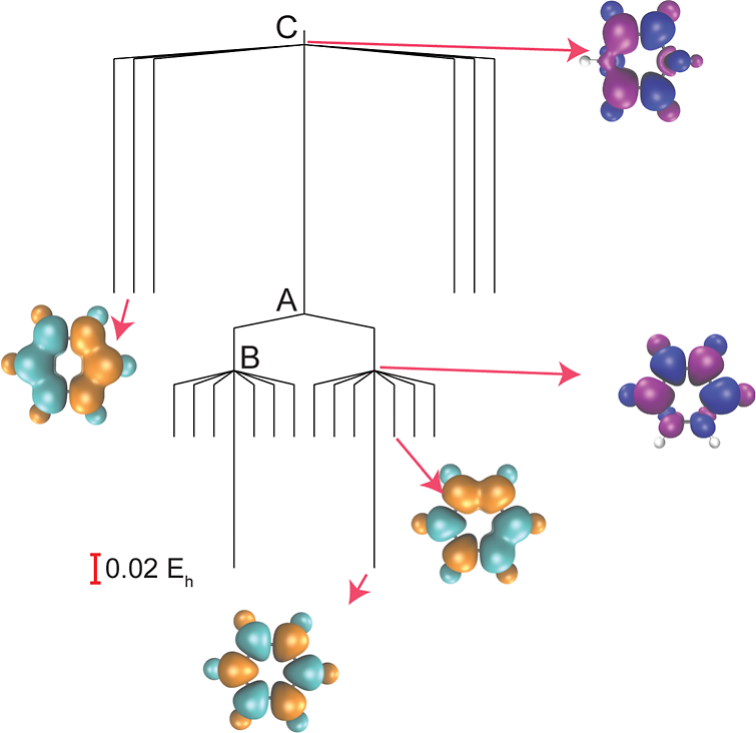
Structure of the SCF energy landscape for benzene (6-31G*) using the PBE correlation functional with 100% PBE exchange and additional 100% HF exchange. Transition state B connects the lowest-energy minimum and the second-lowest-energy minimum. Transition state A connects the lowest-energy minimum with its spin-flip counterpart, and transition state C connects the highest-energy minimum and the global minimum. The transition states illustrate how pairs of local minima are connected by swapping two electrons localized on different atoms, as shown in the selected spin density plots, where a different color scheme has been chosen for the transition states.

**Figure 5:**
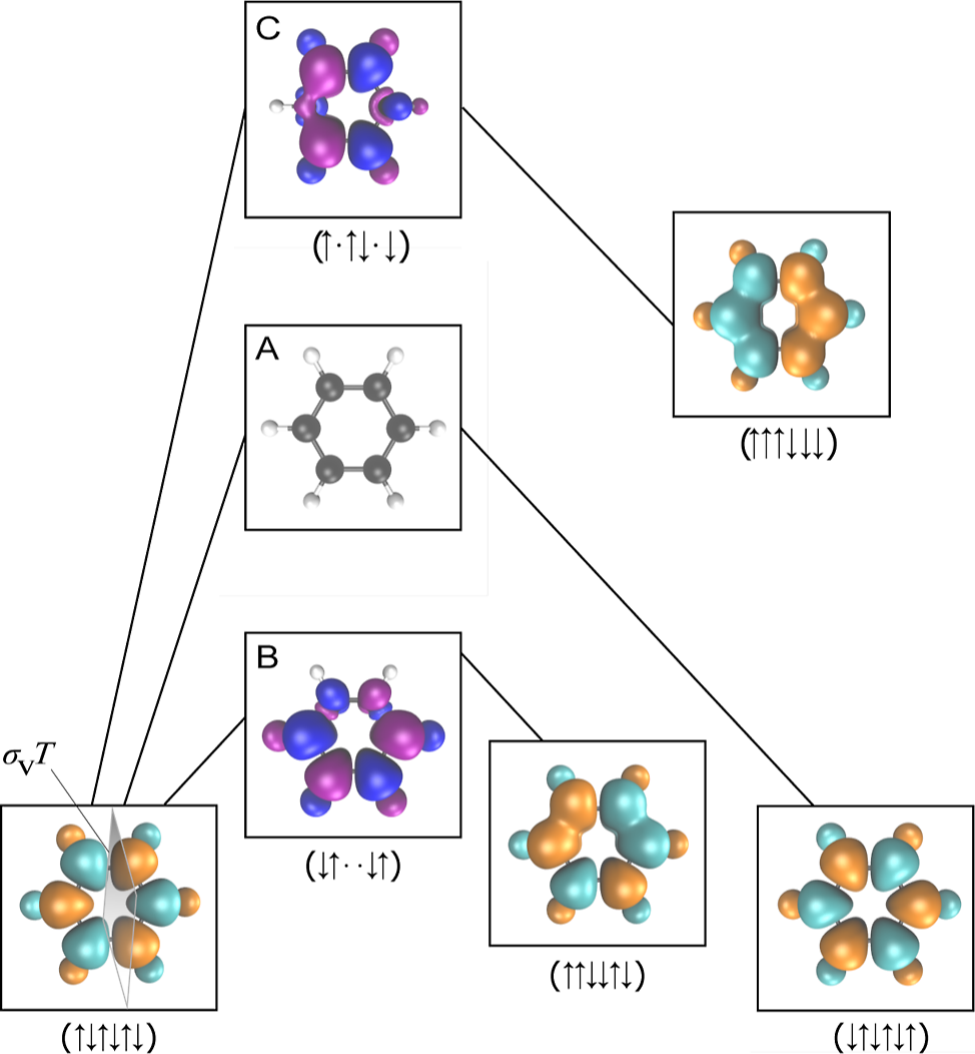
Degenerate lowest-energy minima are connected by a spin-pure transition state (A), which is the global minimum on the RKS energy landscape. The pathway connecting the global minima to the second-lowest-energy minima corresponds to the spin reversal of electrons localized on two neighboring C–H sites (B), while the global minima and highest-energy minima are connected through the reversal of spins on diametrically opposite C–H sites (C). In each case, the symmetry with respect to the combined spatial and time-reversal operation  is preserved along the pathway.

All the local minima in benzene with 100% HF exchange correspond to spin-polarized solutions with the magnitude of the spin density distributed equally among the different carbon out-of-plane p orbitals. The global minimum is still the solution with an antiferromagnetic spin pattern, as seen for pure HF, and these two degenerate global minima are connected by an index-1 saddle point that corresponds to the RHF global minimum (labeled A in Figure 4). The 2-fold degeneracy arises from the global spin-flip operation that interconverts these solutions, and the index-1 saddle point is an RHF solution that conserves this symmetry consistent with the rules for symmetric degenerate rearrangements (Figure 5).^24,59,60^ The next lowest-energy set of local minima forms a 12-fold degenerate set with a different spin polarization that corresponds to the cyclic spin pattern ↑↑↓↓↑↓, while the highest-energy local minima form a 6-fold degenerate set corresponding to the spin pattern ↑↑↑↓↓↓. The degeneracy of each set of local minima is driven by the degree of spatial and spin symmetry breaking in these spin density patterns.

The index-1 saddle points (transition states) provide further insights into the organization of the SCF solution space. In Figures 4 and 5, we illustrate the connectivity between the SCF local minima via index-1 saddle points for benzene. The lowest-energy transition states (B in Figure 4) connect one of the global minima (↑↓↑↓↑↓) to one of the local minima with spin pattern ↑↑↓↓↑↓. We see that these local minima split into two groups of six, with each group forming a local funnel leading to one of the symmetry-broken global minima. Inspecting the spin density of these index-1 saddle points shows that these solutions are formed by swapping the localized electron density on two neighboring atoms, with the saddle point featuring a pair of C–H sites with a reduced spin polarization (Figure 5). Through this mechanism, the spin polarization undergoes the asymmetric transition
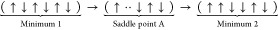
16where “·” denotes a local atomic spin polarization close to 0. Similarly, the highest-energy local minima are connected to a global minimum by an index-1 saddle point (C in Figure 4) where the local spin density is close to zero on two sites that are opposite each other in the ring (Figure 5), showing that these solutions are related by swapping electrons on opposite C–H sites through the mechanism
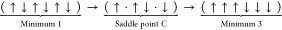
17This analysis again highlights the fundamental insight encoded in both the local minima and connecting pathways on the SCF energy landscape, as previously illustrated for H_4_ isomorphs in ref (24).

**Figure 6:**
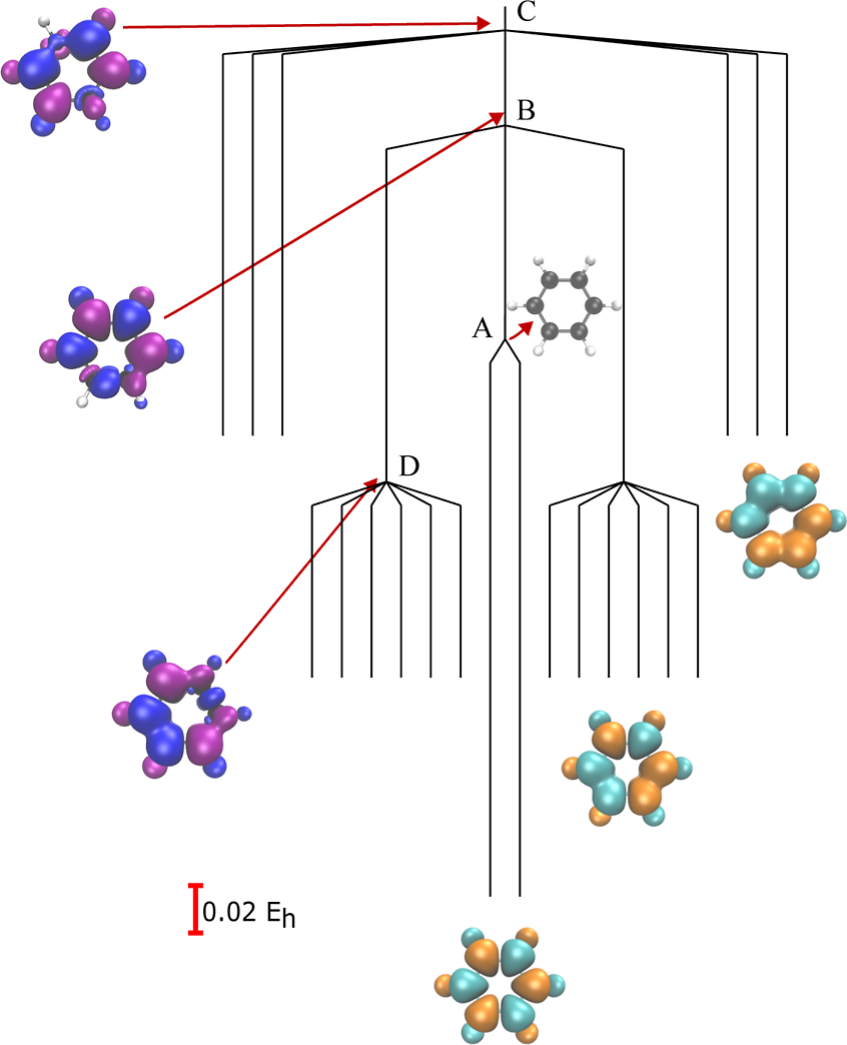
Structure of the SCF energy landscape for benzene (6-31G*) using the B88 exchange functional, LYP correlation functional, and 100% HF exchange. The minima exhibit the same organization and spin density patterns as for the PBE exchange functional, but changes in the ordering of transition states modify the appearance of the graph. In particular, the barrier between the global minima and the ↑↑↓↓↑↓ minima (transition state B) lies above the barrier for the interconversion of the global minima (transition state A), in contrast to the PBE functional.

Finally, we observe that the local minima for benzene with both 100% PBE and 100% HF exchange correspond to all possible allocations of three ↑ and three ↓ electrons on the six carbon atoms in the π system. To check that this result is not specific to the PBE functional, we repeated the calculations for the B88 exchange functional. The resulting landscape, illustrated in Figure 6 for 100% B88 and 100% HF exchange with the LYP correlation functional, has the same organization and degeneracies for the local minima, as shown in Figure 6. However, the energic ordering of transition states changes. Transition state D, which provides a pathway between ↑↑↓↓↑↓ minima, also exists on the PBE landscape, but for PBE, it lies above transition state B. For B88, the corresponding transition state B, connecting the global minimum to the ↑↑↓↓↑↓ set, corresponds to the threshold at which all ↑↓↑↓↑↓ and ↑↑↓↓↑↓ minima can interconvert.

**Figure 7:**
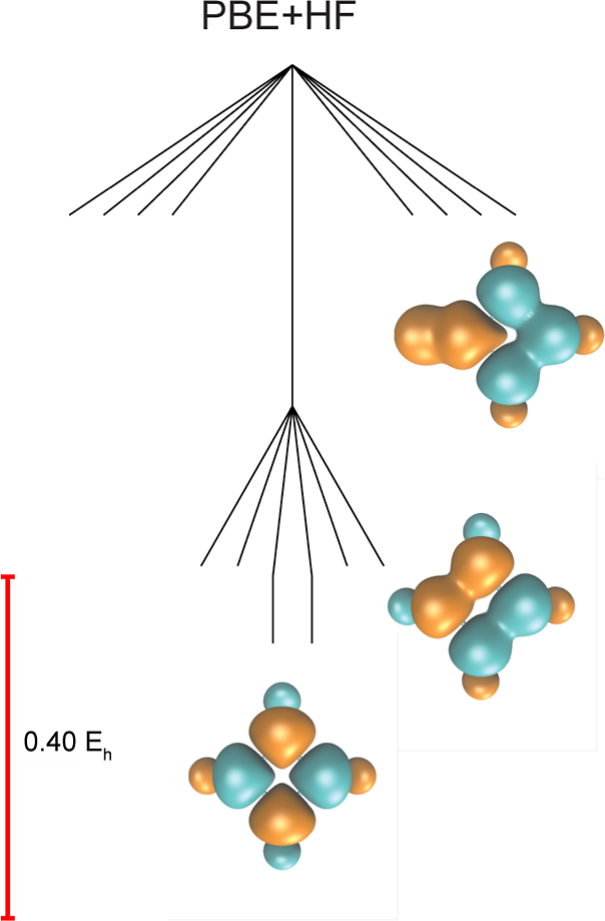
Structure of the SCF energy landscapes for cyclobutadiene (6-31G*) using PBE correlation with both 100% PBE and 100% HF exchange. Including an excess for the exchange contribution produces additional local minima with distinct spin polarization patterns.

These landscapes suggest that the spin symmetry breaking in unsaturated cyclic polyenes with surplus exchange can be understood as a combinatorial problem, with  local minima in total for benzene. Therefore, we predict  local minima for this setup in cyclobutadiene, which has four different sites and two distinct permutational arrangements of the localized spins (corresponding to two degenerate sets of minima). Figure 7 confirms that these local minima can indeed be found for cyclobutadiene with a functional that includes both 100% PBE and 100% HF exchange, and these spin localization patterns have previously been observed for HF with different geometries and basis sets.^61^ Furthermore, we note that this excess exchange can also create local minima with even higher energy, which includes symmetry breaking for the electrons in the C–H bonds in cyclobutadiene (Figure 7). These predictions for the solution landscape of arbitrary cyclic polyenes are particularly interesting and will be investigated further in future work.

### 3.3 Balancing One-Body Delocalization and Exchange Localization

The relative energies of the SCF minima in these cyclic polyenes demonstrate the subtle balance between the pattern of spin polarization and the existence of SCF solutions that break *Ŝ*^2^ symmetry. The results in Figures 4 and 7 show that the number of antiparallel pairs of electrons on neighboring sites decreases as the energy of each local minimum increases. For example, the global minimum in benzene with 100% PBE (or B88) and 100% HF exchange has six antiparallel nearest-neighbor electron pairs, whereas the highest-energy local minimum has only two antiparallel nearest-neighbor pairs. The underlying physics can be elucidated by relating the ground state of cyclic π systems to a cyclic Hubbard model via an extension of the Hückel Hamiltonian. Crucially, the ground state of the Hubbard model in the near-atomic limit is ferromagnetic, since an antiparallel nearest-neighbor electron configuration allows the electrons to delocalize to form local bonding interactions. This one-body energetic stabilization has been derived using second-order perturbation theory in the Hubbard model^62^ and can also be understood through the Pauli exclusion for parallel spins, which prevents the delocalization of parallel spins on neighboring sites. Therefore, although all the local minima in benzene or cyclobutadiene would be almost degenerate if only the two-electron interaction is considered, the number of antiparallel neighboring spin pairs lifts the degeneracy by providing a one-body stabilization through delocalization to form bonding interactions.

These factors provide an explanation for how the balance between one-body effects and two-body exchange interactions affects SCF symmetry breaking. A strong exchange interaction favors the localization of electrons on individual sites, while the one-body delocalization favors nearest-neighbor antiparallel spins. Hence, spin symmetry breaking can be triggered by either increasing the strength of the HF exchange, as we have shown for benzene and cyclobutadiene, or decreasing the strength of one-body interactions.

**Figure 8:**
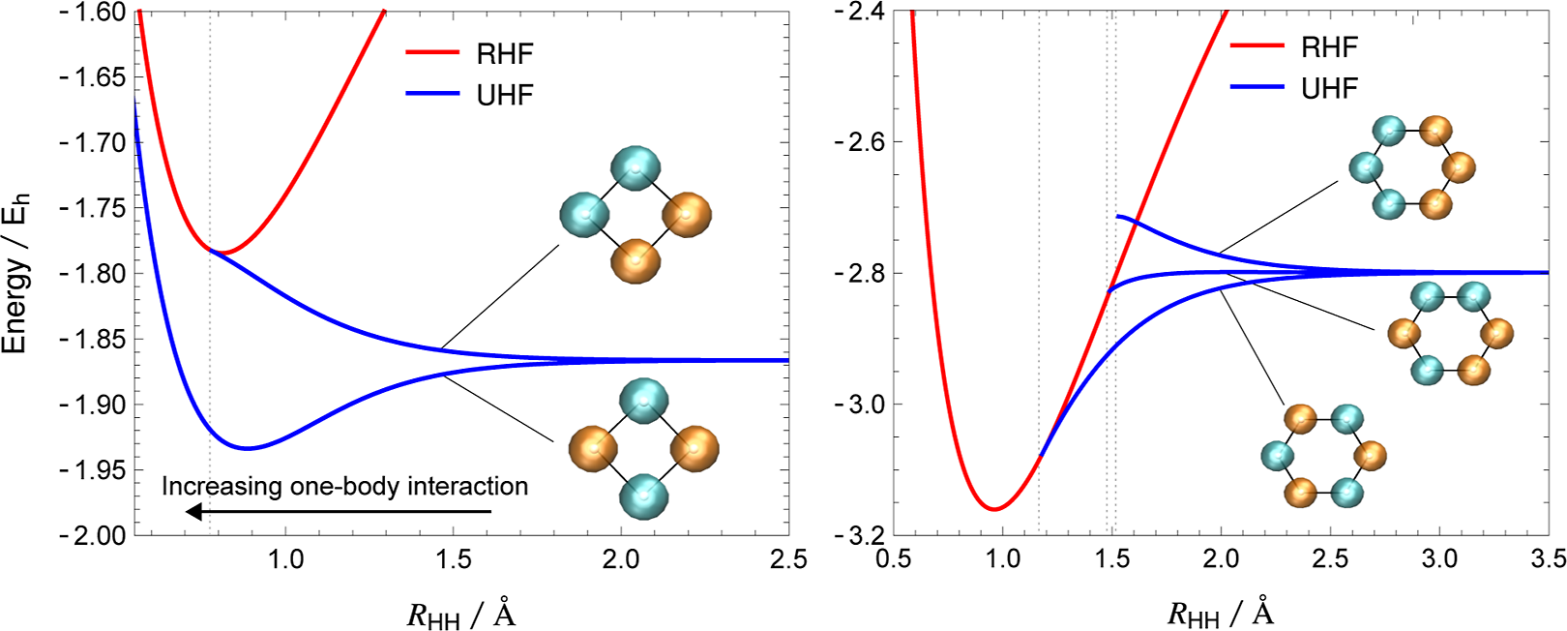
Energies of the UHF local minima in cyclic H_4_ (left) and H_6_ (right) as a function of the H–H bond distance for the STO-3G basis set, with the RHF ground state included for comparison. In the dissociation limit, the UHF minima form spin polarization patterns that are analogous to cyclobutadiene and benzene. As the one-body bonding interaction is turned on at smaller *R*_HH_, the degeneracy is lifted depending on the number of nearest-neighbor antiparallel electrons.

The one-body effect described above can be demonstrated for hydrogen rings in a minimal basis (STO-3G^63^), where the edge length *R*_HH_ controls the strength of bonding delocalization. For cyclic H_4_ and H_6_ at large *R*_HH_, the set of UHF local minima directly mirrors the degeneracies of the cyclobutadiene and benzene solutions for a combined 100% PBE and 100% HF exchange (Figure 8). These solutions are all degenerate in the dissociation limit, but the degeneracy is lifted as the one-body bonding interaction becomes nonzero at shorter bond lengths. For both H_4_ and H_6_, the higher-energy local minima disappear first as *R*_HH_ decreases, consistent with the disappearance of solutions as the importance of the HF exchange contribution decreases (Section 3.2). The bonding interaction eventually becomes so favorable in cyclic H_6_ near equilibrium that the lowest-energy UHF solution coalesces with the RHF ground state and no spin symmetry breaking remains. In contrast, since the RHF state must always include a doubly occupied non-bonding orbital in square H_4_, this weaker one-body interaction means that the symmetry-broken UHF global minimum continues to exist for all bond lengths. Thus, the lack of strong one-body bonding interactions drives the essential UHF symmetry breaking in square H_4_, and we can deduce that the same effect occurs for the diradical character associated with square cyclobutadiene.

## 4 Discussion and Conclusions

The SCF energy landscape intrinsically underpins nearly all electronic structure calculations and determines whether the approach is successful. Remarkably, the structure of these landscapes is still largely unknown, and the rich information encoded in the multiple solutions and their connections is only now starting to be explored and understood. In this contribution, we analyzed the emergence of spin symmetry breaking for the unrestricted SCF energy landscapes of benzene and square cyclobutadiene. We identified spin-polarized global UHF minima for both benzene and cyclobutadiene, in agreement with previous results.^21,61^ For benzene, this symmetry breaking is characterized as artificial and can be removed by including approximate dynamic correlation using the PBE exchange–correlation functional. On the other hand, the symmetry breaking in cyclobutadiene persists with the PBE functional, indicating that this is an essential symmetry breaking that reflects the diradical nature of this molecular structure.

We investigated the relationship between symmetry breaking and the amount of exact HF exchange included in the PBE functional. We found that more spin-polarized solutions emerge when the amount of exact exchange is increased, supporting the findings of Sherrill et al.^13^ Furthermore, introducing surplus exact exchange on top of the PBE exchange functional leads to previously unreported higher-energy local minima that provide additional insights into the role of exchange. These higher-energy solutions encompass all possible spin density patterns for localizing the π electrons in the carbon p orbitals, leading to a combinatorial pattern of local minima, which we expect to generalize to larger even-number cyclic polyenes. This pattern was also observed for analogous calculations with the B88 functional. Examining the pathways that connect different minima reveals that different spin density patterns are related through local spin-flip operations, demonstrating how the fundamental structure of the SCF solution space is encoded in the local minima and saddle points. An extension to larger cyclic polyenes and different charge states would deepen our understanding further, although the potential presence of spin frustration would require generalized approximations.^24,64,65^

Our analysis into the relative energies of local minima with different spin patterns highlights the subtle balance between one- and two-body effects in determining SCF symmetry breaking. In both benzene and cyclobutadiene, the global minima correspond to an antiferromagnetic spin polarization that facilitates bonding interactions by localizing opposite spin electrons on neighboring sites. In contrast, the localization of parallel neighboring spins in higher-energy minima prevents the formation of bonding delocalization through the Pauli exclusion principle. Thus, symmetry breaking can be encouraged by increasing the strength of exchange interactions or by decreasing the one-body interactions. We have further illustrated this principle by analyzing the SCF landscapes on varying the bond length in the cyclic H_4_ and H_6_ analogues of benzene and cyclobutadiene.

Our analysis provides a new perspective on the origins of symmetry breaking in cyclic polyenes such as benzene and square cyclobutadiene. The essential symmetry breaking in cyclobutadiene, which is attributed to diradical open-shell character, can be explained by the weak one-body energy contributions from the nonbonding occupied molecular orbitals. In contrast, the unexpected artificial symmetry breaking in benzene arises from an exaggerated exchange interaction that occurs due to a lack of electron correlation effects. Similar essential symmetry breaking can be expected in transition metal clusters with nearly degenerate weakly bonding orbitals^23^ or larger antiaromatic conjugated systems. The greater nonconvexity of the energy landscape (more local minima) with stronger exact exchange increases the difficulty of global optimization. This effect may operate in systems with open-shell or near-degenerate orbitals, making SCF convergence more challenging in such cases. A better understanding of the fundamental principles that determine the organization of the SCF landscape may help to provide new ways to treat such problems in the future.

## Supporting Information Available

Molecular structure considered for square cyclobutadiene, provided in angstroms (XYZ)
Molecular structure considered for benzene, provided in angstroms (XYZ)

## Supplementary Material

ct4c01404_si_001.xyz

ct4c01404_si_002.xyz
